# Evaluation of ChatGPT's Capabilities in Medical Report Generation

**DOI:** 10.7759/cureus.37589

**Published:** 2023-04-14

**Authors:** Zeyu Zhou

**Affiliations:** 1 Computer Science, Georgia Institute of Technology, Atlanta, USA

**Keywords:** gerd, epigastric pain, chatgpt, epigastric, medical examination

## Abstract

The growing demand for efficient healthcare delivery has intensified the need for technological innovations that facilitate medical professionals' decision-making processes. In this study, we investigate ChatGPT (OpenAI Incorporated, Mission District, San Francisco, United States), a state-of-the-art language model based on the GPT-4 architecture, as an effective tool for assisting healthcare professionals in writing medical reports based on real patient laboratory results. By leveraging ChatGPT's extraordinary performance across multiple medical domains, including lab result diagnostics and medical literature analysis, we aimed to streamline and enhance the medical report generation process.

The generated case report presents a 31-year-old male patient with no significant past medical history who visited a clinic to establish care and seek evaluation for abdominal pain. Following routine laboratory tests, including a complete blood count, comprehensive metabolic panel, and a Helicobacter pylori breath test, ChatGPT provided tailored recommendations addressing identified concerns and abnormalities. These included lifestyle modifications, such as dietary changes, weight management, and avoiding trigger foods or behaviors; alongside medical treatment options, the patient was advised to consult a gastroenterologist for further evaluation and potential advanced treatment options.

The organization and structure of this case study are derived from ChatGPT's output, using patient's actual physical information and lab results as input, without any prior knowledge. Ultimately, we will compare the generated report with suggestions from an online doctor consultation system to demonstrate the precision and reliability of ChatGPT's recommendations. Through this comparison, we aim to show that ChatGPT can produce coherent, comprehensive, and clinically relevant medical reports with a relatively high degree of accuracy and consistency.

## Introduction

The rapid advancements in artificial intelligence (AI) and natural language processing (NLP) technologies have led to the emergence of sophisticated language models capable of imitating human-like text generation [1,2,3]. ChatGPT (OpenAI Incorporated, Mission District, San Francisco, United States), a state-of-the-art language model based on the GPT-4 architecture, has been trained on extensive volumes of internet text and has demonstrated exceptional performance in various roles within healthcare and health research. Since its initial public access release in November 2022, subsequent versions such as GPT-4 have been increasingly equipped with AI-guided conversation (AIGC) capabilities. This advancement has enabled ChatGPT to assist doctors in tasks such as initial patient assessments, disease diagnosis, and treatment suggestions. In March 2023, Meta introduced LLaMA (Large Language Model Meta AI) [4], which was followed by the proposal and development of a specialized AI doctor model called ChatDoctor [5]. This new model, ChatDoctor, was trained using real patient-doctor conversations collected from an online Q&A medical consultation platform iCliniq (icliniq.com). When compared to ChatGPT's accuracy of 87.5%, ChatDoctor demonstrated a higher accuracy rate, achieving 91.25% on average.

In this case report, we explore the potential of ChatGPT to provide clinically relevant and accurate medical texts that can pass the Turing test, which measures a machine's ability to exhibit human-like intelligence. We focus on a real-world example of gastroesophageal reflux disease (GERD) with actual laboratory results to evaluate ChatGPT's capacity to generate recommendations for patients seeking medical advice from AI-based platforms. Our primary objective is to develop a rapid and relatively accurate approach to harness the power of AI in guiding patient care, ultimately improving healthcare delivery and enhancing patient outcomes. At the end of the study, we will use the same patient case on iCliniq (icliniq.com) and MedicalChat (medical.chat-data.com) as reference points to evaluate how well ChatGPT's generated medical report compares to the solutions provided by these online doctor consultation platforms. Additionally, we will include the Zero-GPT testing results as a benchmark to demonstrate the human-like quality of the generated text, showcasing its ability to pass the Turing test [6].

Epigastric pain is a common complaint among patients seeking medical care [7-14]. The causes of epigastric pain are numerous and can range from benign conditions to life-threatening emergencies [11]. Identifying the underlying cause of epigastric pain is essential for appropriate management and treatment [10]. The esophagus is a muscular tube that moves food from the mouth to the stomach [9]. It is equipped with a valve called the lower esophageal sphincter that prevents stomach acid from flowing upward [12,13]. However, when this valve fails, stomach contents, including acid, can reflux into the esophagus [12]. This condition is known as GERD [12,13], which can cause irritation, pain, difficulty swallowing or breathing, and in severe cases, recurring pneumonia from aspirating stomach contents [13]. GERD is a condition caused by excessive backflow of stomach acid into the esophagus. This occurs when the lower esophageal sphincter, a muscle that acts as a valve between the esophagus and stomach, weakens or relaxes inappropriately, allowing stomach contents, including acid, to reflux into the esophagus. In recent years, there has been an increase in the number of patients seeking medical treatment for GERD. Estimations by El-Serag suggest that the prevalence of GERD in the United States ranges from 18.1% to 27.8% [14]. Although GERD has been investigated as a global health issue, research in industrialized countries tends to focus on individual cases or specific populations. Symptoms of GERD include burning, pressure or sharp pain in the upper abdomen or mid-to-lower chest, belching, acid taste in the back of the throat, chronic cough, sore throat, and hoarseness. Symptoms can occur after meals, particularly large ones, and at night when lying down [12,13].

## Case presentation

ChatGPT is capable of tokenizing input words and generating properly formatted outputs even when the input is messy or contains visible errors. This capability allows it to understand and interpret imperfect user inputs, providing more accurate and coherent responses. To begin generating the case presentation using ChatGPT, we first need to provide a detailed description of the patient's symptoms and lab results as input. After receiving ChatGPT's initial response, we should organize and refine the sentences to ensure clarity and semantical consistency. Next, we can ask ChatGPT to analyze the results and provide suggestions based on the patient's symptoms and medical history.

**Figure 1:**
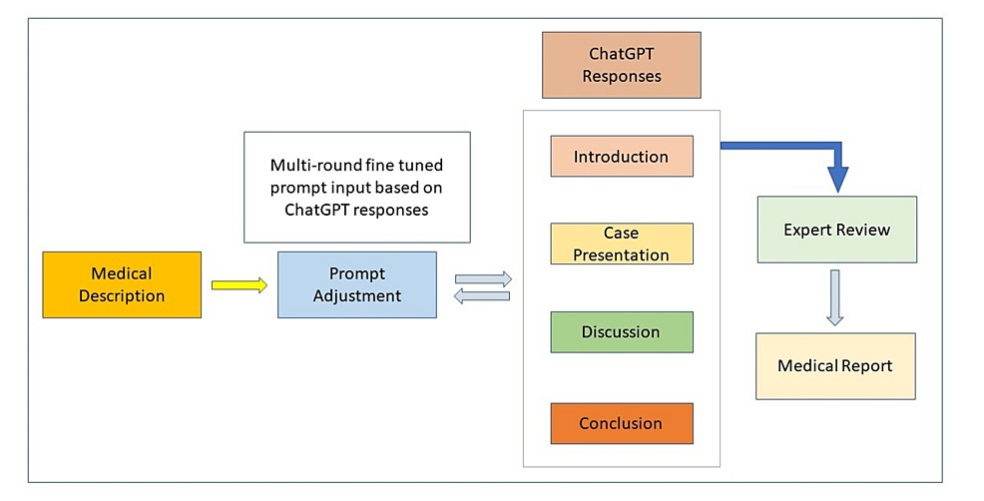
Medical report generation workflow

Figure 1 illustrates a typical workflow for a medical report generation pipeline, where we utilize ChatGPT to create case presentations, discussions, and conclusions.

To ensure compliance with the ChatGPT's 4096-tokens limitation, the input medical texts are structured into several parts as follows:

"31-year-old male patient with no significant past medical history who presented to a clinic to establish care. Routine labs, including a CBC, CMP, and H pylori breath test, were ordered while the patient was fasting. after reading lab results, proper suggestions have been made to the patients. First visit with physical exam, and second visit with lab results check. Suggestion taking VitaD and good food eating habit. After 6 month remote checking, he felt quite good then*."*

"the patient states to be currently taking Medication Sig Ascorbic Acid (VITAMIN C) 100 MG tablet Take 100 mg by mouth daily fish oil 1000 mg capsule Take 1 g by mouth three (3) times daily. Blood pressure: 125/82, BMI: 25.40, Weight: 177 lb, Height: 5'10", Temperature: 97.3 °F, Pulse: 75, Respiration: 16, Oxygen saturation: 98%*."*

"First visit, physical exam, General Appearance: Well-developed and well nourished. Head/Eyes/Neck/Mouth/Throat: Normocephalic and atraumatic. External ear normal, canal normal, and TM-good light reflex. Nose normal. Oropharynx is clear and moist, no oral lesions, dentition within normal. Conjunctivae and EOM are normal. Pupils are equal, round, and reactive to light. Normal range of motion. Neck supple. No thyromegaly present, no thyroid nodules, no bruits, no adenopathy. Respiratory System: Effort, and normal breath sound. No stridor or respiratory distress was noted. No wheezes, rhonchi, or rales were present. Cardiovascular: Normal rate, regular rhythm, normal heart sounds and intact distal pulses. Exam reveals no murmur. Gastrointestinal System: Soft abdomen, and normal bowel sounds. No distension, masses, hepatosplenomegaly, tenderness, rebound, or guarding was noted. Genitourinary: No CVA tenderness. Lymphatic: No abnormalities noted. Musculoskeletal: Normal range of motion. No edema and no tenderness. Skin: Skin is warm and dry. No rash noted. No erythema. No pallor. Neurological System: The patient was alert and oriented to person, place and time. CN grossly intact. Muscle tone was normal. Psychiatric System: Normal mood and affect. Judgment and thought content normal"

"Immunization History. Administered Date(s) Administered. COVID-19, mRNA, (Pfizer - Purple Cap) 30 mcg/0.3 mL 04/17/2021, 04/17/2021, 05/15/2021, 05/15/2021, 12/16/2021."

"Second visit, lab results CBC W/ AUTO DIFFERENTIAL - Details, format Component, Your Value, Standard Range, Flag: WBC, 6.6 10*3/uL, 4.0 - 10.0 10*3/uL; RBC 4.90 10*6/uL 4.04 - 5.75 10*6/uL, Please note new RBC reference range effective 11/2/21, Hemoglobin 15.1 g/dL 13.0 - 17.0 g/dL, Hematocrit 45.0 % 38.8 - 51.0 % Please note new Hematocrit reference range effective 11/2/21, MCV 91.8 fL 82.4-100.9 fL fL Please note new MCV reference range effective 11/2/21 MCH 30.8 pg 26.0 - 34.0 pg MCHC 33.6 g/dL 30.0 - 36.0 g/dL RDW-CV 12.2 % 11.6 - 14.4 % RDW-SD 41.7 fL 35.6 - 48.0 fL Platelet Count 266 10*3/uL 150 - 450 10*3/uL MPV 9.3 fL 9.4 - 12.4 fL L Neutrophils Relative 67 % % Lymphocytes Relative 26 % % Monocytes Relative 5 % % Eosinophils Relative 1 % % Basophils Relative 1 % % Immature Granulocytes % 0 % % Neutrophils Absolute 4.4 10*3/uL 1.7 - 6.4 10*3/uL Lymphocytes Absolute 1.7 10*3/uL 1.0 - 3.5 10*3/uL Monocytes Absolute 0.4 10*3/uL 0.1 - 0.8 10*3/uL Eosinophils Absolute 0.0 10*3/uL 0.0 - 0.4 10*3/uL Basophils Absolute 0.0 10*3/uL 0.0 - 0.2 10*3/uL Absolute Immature Granulocytes 0.0 10*3/uL 0.0 - 0.2 10*3/uL Nucleated RBCS 0.0 /100 WBCs <1.0 /100 WBCs"

"Second visit, lab results COMPREHENSIVE METABOLIC PANEL - Details, organize the table: Component, Your Value, Standard Range, Flag; Sodium 136 mmol/L 134 - 145 mmol/L, Potassium 3.9 mmol/L 3.5 - 5.1 mmol/L, Chloride 101 mmol/L 98 - 110 mmol/L, CO2 28 mmol/L 21 - 32 mmol/L, Anion Gap 7 mmol/L 3 - 14 mmol/L Blood Urea Nitrogen (BUN) 13 mg/dL 7 - 24 mg/dL, Creatinine 0.74 mg/dL 0.70 - 1.30 mg/dL, eGFR 123 mL/min/1.73m2 >60 mL/min/1.73m2, BUN/Creatinine Ratio 17.6 7.0 - 28.0, Calcium 9.3 mg/dL 8.2 - 10.2 mg/dL, Total Protein 8.4 g/dL 6.4 - 8.2 g/dL H, Albumin 4.3 g/dL 3.4 - 5.0 g/dL, Globulin 4.1 g/dL 2.0 - 4.1 g/dL, Albumin/Globulin Ratio 1.0 0.9 - 1.8, Total Bilirubin 1.0 mg/dL 0.2 - 1.0 mg/dL, AST (SGOT) 21 U/L<50 U/L, ALT (SGPT) 28 U/L<61 U/L, Alkaline Phosphatase 92 U/L, 42 - 122 U/L Fasting 8 hours or more? Yes, Glucose 93 mg/dL, 75 - 99 mg/dL, Normal fasting, 75-99 mg/dL, Impaired fasting, 100-125 mg/dL, Provisional diabetic, fasting over 125 mg/dL, Non fasting, 75-140 mg/dL"

"Second visit, lab results 25-OH VITAMIN D, Component, Your Value, Standard Range, Flag, 25-OH Vitamin D, Total 10 ng/mL 30 - 100 ng/mL L Deficiency < 20 ng/mL Insufficiency 20 to 29 ng/mL Sufficiency 30 to 100 ng/mL Possible Toxicity > 100 ng/mL"

After several rounds of conversational fine-tuning, we have obtained the following descriptive case presentation:

The 31-year-old male patient, who had experienced intermittent epigastric pain over the past several weeks, tried to establish medical care and seek evaluation for abdominal pain. The patient reported experiencing significant mental stress from work and disordered eating habits, which were believed to contribute to the symptoms. In addition to epigastric pain, the patient had been experiencing heartburn. The patient observed that the condition improved when stress levels were reduced if he maintained regular eating patterns.

In the first clinic visit, the patient was asymptomatic and did not present any additional symptoms or abnormalities upon physical examination. The patient had no history of drug abuse. The patient is currently taking two medications: ascorbic acid (vitamin C) 100 mg tablet, taken orally once a day, and fish oil 1000 mg capsule, taken orally three times a day (3 g total per day) (Table 1).

**Table 1:** Patient's medicines

Medication	Dosage	Frequency	Route
Ascorbic acid	100 mg tablet	Once a day	Oral
Fish oil	1000 mg capsule	Three times a day (3 g total per day)	Oral

The patient's vital signs were as follows: blood pressure 125/82 mmHg, BMI 25.40, weight 177 lbs, height 5'10", temperature 97.3°F, pulse 75 bpm, respiration rate 16 breaths per minute, and oxygen saturation at 98%. During the physical examination, the patient appeared well-developed and well-nourished, with some signs related to GERD. The head, eyes, neck, mouth, and throat examination revealed mild erythema in the oropharynx, possibly associated with reflux. The respiratory and cardiovascular systems were unremarkable, with normal breath sounds, heart rate, rhythm, and no murmurs. The gastrointestinal system exhibited a soft abdomen with normal bowel sounds, but mild epigastric tenderness was noted upon palpation, which may suggest GERD. Genitourinary examination showed no costovertebral angle tenderness. The lymphatic system was normal, and the musculoskeletal system had a normal range of motion without edema or tenderness. The patient's skin was warm, dry, and free of rashes or erythema. Neurologically, the patient was alert and oriented, and had intact cranial nerves and normal muscle tone. The psychiatric evaluation revealed a normal mood, affect, judgment, and thought content.

The patient received the COVID-19 immunization with the mRNA Pfizer vaccine (purple cap) at a dosage of 30 mcg/0.3 mL on four occasions, with administration dates of 04/17/2021, 05/15/2021, and 12/16/2021. The patient reported no side effects following the vaccinations, indicating a well-tolerated immunization experience. The vaccine is not considered to be related to the patient's epigastric pain and heartburn symptoms.

During the patient's second visit, fasting laboratory tests were ordered and evaluated to further investigate their symptoms. These tests included a complete blood count (CBC) (Table 2), a comprehensive metabolic panel (CMP) (Table 3), a Helicobacter pylori breath test, and vitamin D (Table 4). The purpose of these tests was to help identify any underlying conditions or abnormalities that could be contributing to the patient's intermittent epigastric pain, heartburn, and other related symptoms.

**Table 2:** Details of CBC W/ auto differential CBC: Complete blood count; WBC: white blood cell; RBC: red blood cell; MCV: mean corpuscular volume; MCH: mean corpuscular hemoglobin; MCHC: mean corpuscular hemoglobin concentration; RDW-CV: red cell distribution width-corpuscular volume; RDW-SD: red cell distribution width-standard deviation; MPV: mean platelet volume:

Component	Your Value	Standard Range	Flag
WBC	6.6 10*3/μL	4.0 - 10.0 10*3/μL	
RBC	4.90 10*6/μL	4.04 - 5.75 10*6/μL	
Hemoglobin	15.1 g/dL	13.0 - 17.0 g/dL	
Hematocrit	45.00%	38.8 - 51.0 %	
MCV	91.8 fL	82.4-100.9 fL	
MCH	30.8 pg	26.0 - 34.0 pg	
MCHC	33.6 g/dL	30.0 - 36.0 g/dL	
RDW-CV	12.20%	11.6 - 14.4 %	
RDW-SD	41.7 fL	35.6 - 48.0 fL	
Platelet Count	266 10*3/μL	150 - 450 10*3/μL	
MPV	9.3 fL	9.4 - 12.4 f	L
Neutrophils Relative	67%	%	
Lymphocytes Relative	26%	%	
Monocytes Relative	5%	%	
Eosinophils Relative	1%	%	
Basophils Relative	1%	%	
Immature Granulocytes %	0%	%	
Neutrophils Absolute	4.4 10*3/μL	1.7 - 6.4 10*3/μL	
Lymphocytes Absolute	1.7 10*3/μL	1.0 - 3.5 10*3/μL	
Monocytes Absolute	0.4 10*3/μL	0.1 - 0.8 10*3/μL	
Eosinophils Absolute	0.0 10*3/μL	0.0 - 0.4 10*3/μL	
Basophils Absolute	0.0 10*3/μL	0.0 - 0.2 10*3/μL	
Absolute Immature Granulocytes	0.0 10*3/μL	0.0 - 0.2 10*3/μL	
Nucleated RBCs	0.0 /100 WBCs	<1.0 /100 WBCs	

**Table 3:** Details of the comprehensive metabolic panel *Total Bilirubin: Use of this assay is not recommended for patients undergoing treatment with eltrombopag due to the potential for falsely elevated results. **Glucose: Normal fasting, 75-99 mg/dL, impaired fasting, 100-125 mg/dL, provisional diabetic, fasting over 125 mg/dL, and non-fasting, 75-140 mg/dL. BUN: Blood area nitrogen; eGFR: estimated glomerular filtration rate; AST: aspartate aminotransferase; ALT: alanine transaminase

Component	Your Value	Standard Range	Flag
Sodium	136 mmol/L	134 - 145 mmol/L	
Potassium	3.9 mmol/L	3.5 - 5.1 mmol/L	
Chloride	101 mmol/L	98 - 110 mmol/L	
CO_2_	28 mmol/L	21 - 32 mmol/L	
Anion Gap	7 mmol/L	3 - 14 mmol/L	
BUN	13 mg/dL	7 - 24 mg/dL	
Creatinine	0.74 mg/dL	0.70 - 1.30 mg/dL	
eGFR	123 mL/min/1.73m2	>60 mL/min/1.73m2	
BUN/Creatinine Ratio	17.6	7.0 - 28.0	
Calcium	9.3 mg/dL	8.2 - 10.2 mg/dL	
Total Protein	8.4 g/dL	6.4 - 8.2 g/dL	H
Albumin	4.3 g/dL	3.4 - 5.0 g/dL	
Globulin	4.1 g/dL	2.0 - 4.1 g/dL	
Albumin/Globulin Ratio	1.0	0.9 - 1.8	
Total Bilirubin*	1.0 mg/dL	0.2 - 1.0 mg/dL	
AST (SGOT)	21 U/L	<50 U/L	
ALT (SGPT)	28 U/L	<61 U/L	
Alkaline Phosphatase	92 U/L	42 - 122 U/L	
Fasting 8 Hours or More?	Yes		
Glucose**	93 mg/dL	75 - 99 mg/dL	

**Table 4:** Details of 25-OH vitamin D, total

Component	Your Value	Standard Range	Flag
25-OH Vitamin D, Total	10 ng/mL	30 - 100 ng/mL	L (Deficiency < 20 ng/mL, Insufficiency 20 to 29 ng/mL, Sufficiency 30 to 100 ng/mL, Possible Toxicity > 100 ng/mL)

The patient's CBC results are presented in Table 2, showing values within the standard range for white blood cells (WBCs), red blood cells (RBC), hemoglobin, hematocrit, mean corpuscular volume (MCV), mean corpuscular hemoglobin (MCH), mean corpuscular hemoglobin concentration (MCHC), red cell distribution width (RDW-CV and RDW-SD), platelet count, and mean platelet volume (MPV). The differential blood count reveals the percentages and absolute values of neutrophils, lymphocytes, monocytes, eosinophils, basophils, and immature granulocytes, which are all within normal limits. Nucleated RBCs are less than 1.0 per 100 WBCs, also within the standard range.

Several interventions were recommended for the patient, including consuming vitamin D-rich foods, taking vitamin D supplements, adhering to the prescribed omeprazole regimen, implementing stress reduction techniques, and making lifestyle modifications. Ongoing monitoring and periodic follow-up visits will serve to support the patient's continued progress and address any potential concerns that may emerge in the future.

Six months after the second visit, during a remote follow-up with the patient, it was noted that his symptoms and overall well-being had shown considerable improvement. This positive development could be attributed to a combination of factors such as appropriate medical intervention, lifestyle modifications, and the natural progression of their condition.

From the generated descriptions, it can be seen that ChatGPT is capable of creating a comprehensive narrative about patients' full medical history and medical situation based on lab results. Additionally, the model provides medical advice rooted in its knowledge base and offers a summary of the patient's current health status.

## Discussion

GERD is a digestive disorder characterized by the frequent backflow of stomach acid into the esophagus [12,13]. This reflux can cause irritation and damage to the esophageal lining, leading to symptoms such as heartburn, regurgitation, and difficulty in swallowing [13]. Various factors, including diet, lifestyle, and genetics, can contribute to the development of GERD [12,13].

The patient's lifestyle questionnaire indicated that he was experiencing significant mental stress from work and irregular eating habits. This information suggests that stress and irregular eating habits may be contributing to the patient's epigastric pain symptoms. It is essential for the patient to address these factors, as lifestyle modifications can significantly improve the conditions. Incorporating stress reduction techniques and adopting healthier eating habits may help alleviate the patient's symptoms and improve their overall well-being.

The patient's annual physical exam also revealed mild anxiety and moderate major depression related to work. Although the patient declined medication or behavioral health assistance, the physician addressed the patient's mental health concerns. In addition, the patient was found to have unspecified hyperlipidemia, for which lifestyle modifications were recommended. 

These laboratory test results outline the patient's general health situation. The patient's HbA1c level is within the standard range, indicating satisfactory long-term blood sugar control. The hepatitis C antibody test is negative, signifying no exposure to the hepatitis C virus (Table 2). The Hep Bs antigen test is negative, showing no current hepatitis B infection, but the Hep Bs antibody test is positive, indicating either vaccination or previous infection and immunity development. The patient's Vitamin B12 level falls within the normal range, ensuring adequate levels of this essential vitamin (Table 3). The Helicobacter pylori breath test is negative, suggesting no current Helicobacter pylori infection, which can cause gastritis and peptic ulcers. However, the patient has a vitamin D deficiency, which can negatively impact bone and overall health (Table 4). Prescription of cholecalciferol 1250 mcg capsules is suggested to be taken once a week for eight doses, with a follow-up vitamin D level check afterward. The patient reported experiencing epigastric pain, which was diagnosed as likely GERD. To manage the condition, the patient was advised to make dietary changes and prescribed omeprazole 40 mg delayed-release capsules to be taken daily. As per healthcare professional's guidance, adjustments to the patient's diet were made by incorporating vitamin D-rich foods such as fatty fish, egg yolks, and fortified dairy products. Additionally, the patient was encouraged to engage in safe sun exposure practices to facilitate natural vitamin D synthesis. The patient was also instructed to follow up with a gastroenterology specialist if the symptoms persist or worsen.

To further evaluate the coherence and precision of the generated medical texts, we also uploaded the actual patient's medical history and symptom descriptions to online doctor consultation websites MedicalChat and iCliniq. By comparing the ChatGPT-generated medical report with the feedback and recommendations provided by medical professionals on the consultation website, we can gain valuable insights into the accuracy and clinical relevance of the generated medical texts under the given topic. 

**Figure 2:**
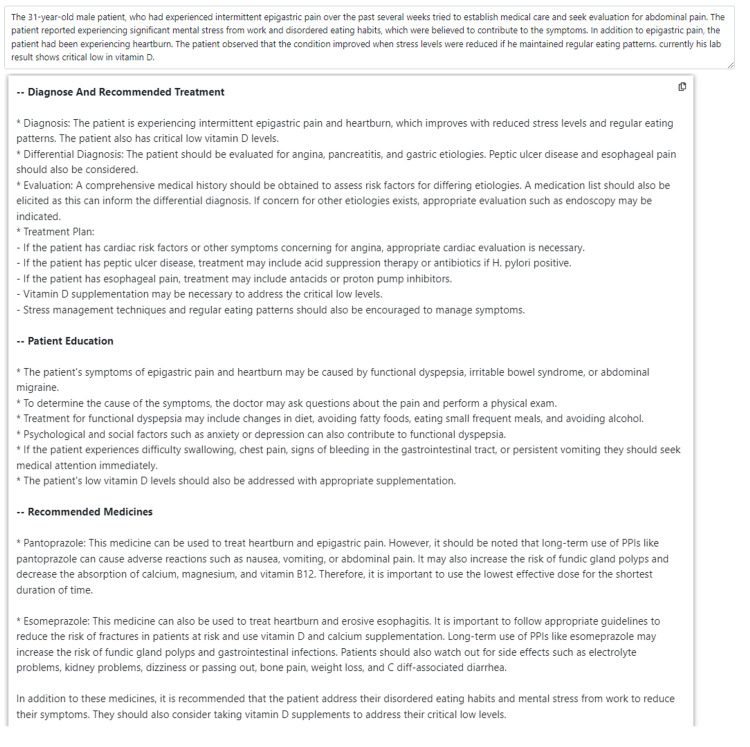
MedicalChat's medical suggestions

Upon reviewing the MedicalChat results (Figure 2), the system recommended taking vitamin D for deficiency issues and the use of pantoprazole and esomeprazole to treat heartburn and epigastric pain.

**Figure 3:**
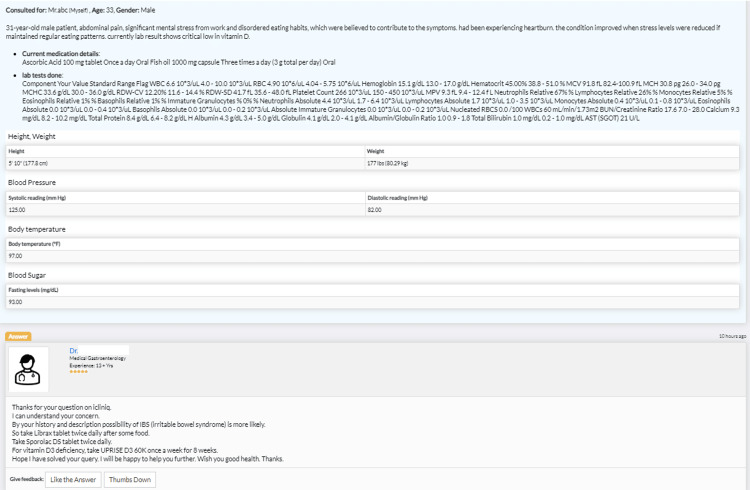
iCliniq's medical suggestions

Upon reviewing the iCliniq results (Figure 3), the doctor also recommended the intake of vitamin D supplements and the use of Sporolac DS tablets to alleviate reflux symptoms. We have properly removed online doctors' information.

In this specific case, where we have access to the actual patient outcome, we can make comparisons. This comparison will help evaluate the accuracy and relevance of the medical information provided by ChatGPT in relation to its counterparts. Based on the comparison of results from ChatGPT, iCliniq, and MedicalChat, it can be concluded that ChatGPT is capable of providing medical suggestions. ChatGPT's suggested solutions closely align with those provided by real doctors, as long as quantified prescriptions are not taken into consideration.

## Conclusions

In conclusion, the patient's laboratory test results, physical examination findings, and lifestyle factors suggest that GERD may be the primary cause of their intermittent epigastric pain and heartburn. The patient's reported improvement in symptoms with stress reduction and regular eating patterns further supports this diagnosis. A comprehensive, patient-centered approach that includes lifestyle modifications, stress reduction techniques, and regular monitoring can significantly improve the management of GERD and enhance the patient's overall well-being. This case report demonstrates the importance of a thorough assessment and evidence-based recommendations in providing effective, personalized care for patients with GERD.

The patient, who was deficient in vitamin D, experienced severe mental stress from work, had disordered eating habits, and suffered from esophageal pain, benefitted significantly from targeted interventions that complied with GERD treatment guidelines. By following the suggestions of taking vitamin D supplements, daily taking omeprazole 40 mg delayed-release capsules, and adopting regular dietary habits, the patient's general health was restored, and his GERD symptoms were managed effectively.

The above two paragraphs, generated by ChatGPT, provide a conclusion for this medical case. They make a summary of the patient's situation, case evaluation, and appropriate medical interventions. Earlier we utilized ChatGPT to generate and format the case presentation section, along with tabular data; the language model provided necessary analysis of lab results and offered possible explanations for the medical case based on the given information. However, ChatGPT currently lacks multimodal processing capabilities and cannot interpret typical X-rays, MRIs, or other medical images. As a result, the entire case report relies solely on textual medical input. If additional medical imaging analysis or interpretation is required, extra work or an alternative approach would be necessary. Moreover, medical prescriptions still require human intervention due to ethical considerations; ChatGPT cannot provide quantitative suggestions on medication details. This limitation may be addressed in future releases with proper fine-tuning and the incorporation of more appropriate medical training data.

We also conducted a test using Zero-GPT, another AI language model, to evaluate the AI similarity of the generated medical report. The results provided in the appendix indicate that it is challenging to discern that the report was helped written by a machine. This observation highlights the advanced capabilities of ChatGPT in generating human-like, coherent, and contextually relevant medical reports. These test results may vary over time as more training data is incorporated into Zero-GPT.

Above all, ChatGPT has shown its potential to assist healthcare professionals in medical report writing. By leveraging this state-of-the-art language model, healthcare providers can optimize their time and resources, allowing them to focus on critical aspects of patient care. As ChatGPT continues to evolve and improve, its applications in the healthcare sector are expected to expand, ultimately contributing to more efficient and patient-centered care delivery.

## Figures and Tables

**Figure 4:**
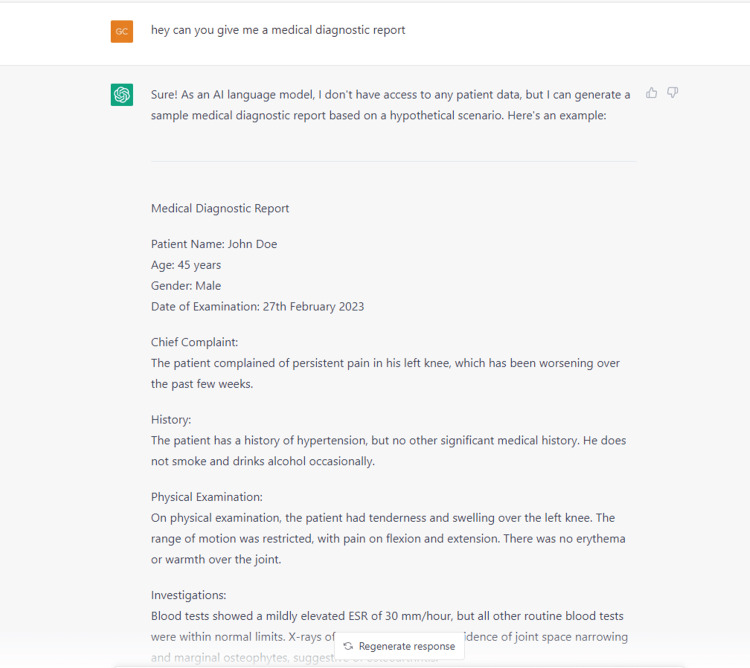
ChatGPT generation of medical report structure

**Figure 5:**
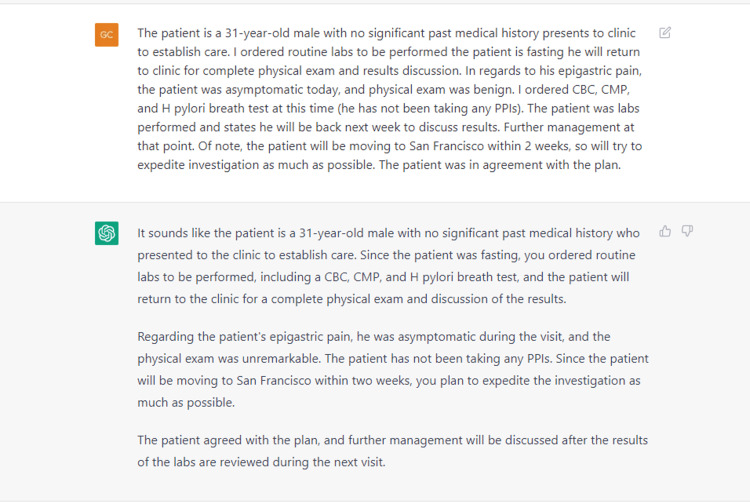
ChatGPT dialogue transcript-1

**Figure 6:**
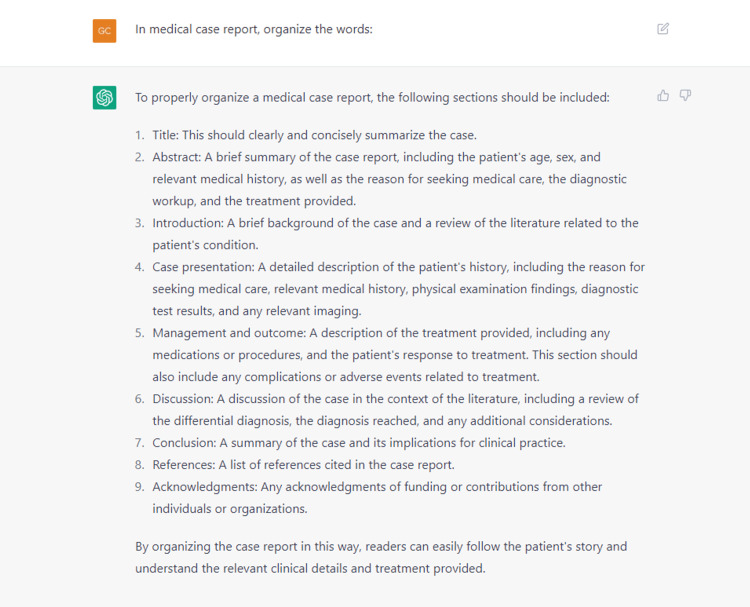
ChatGPT dialogue transcript-2

**Figure 7:**
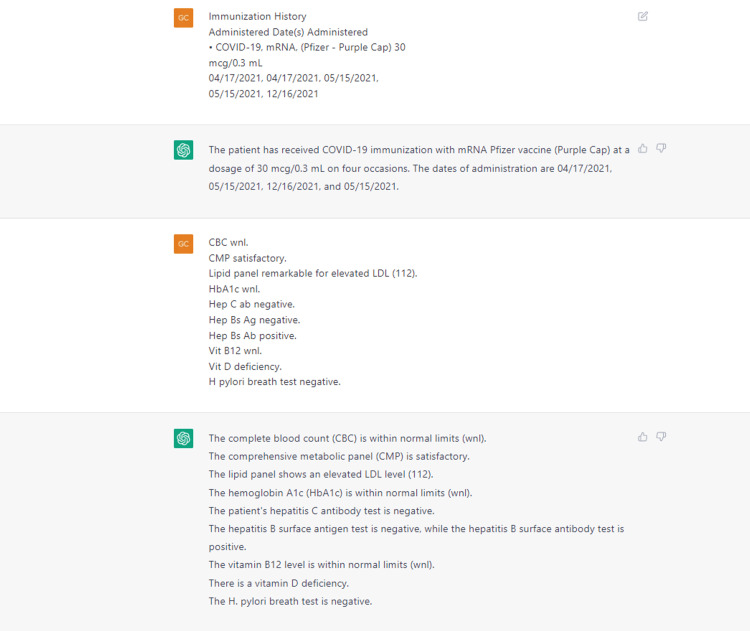
ChatGPT dialogue transcript-3

**Figure 8:**
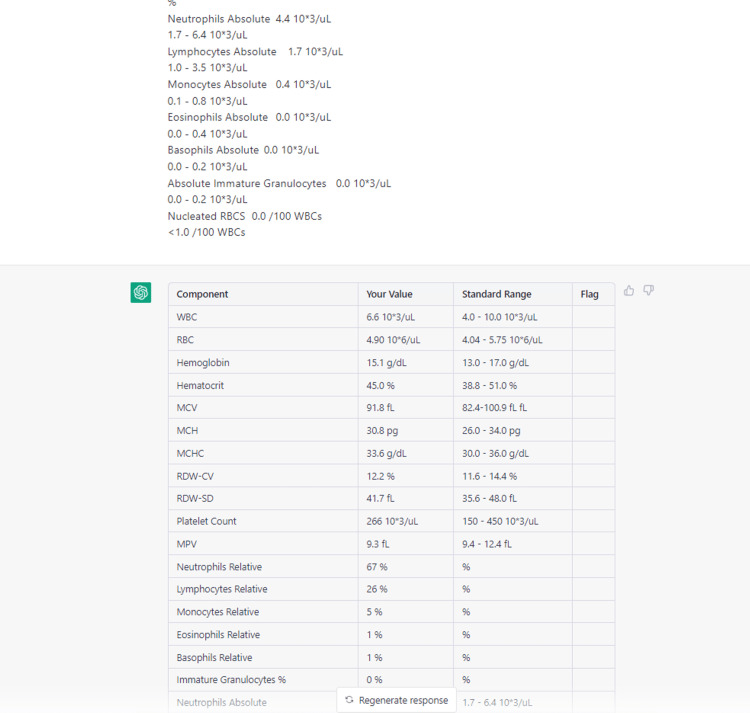
ChatGPT dialogue transcript-4

**Figure 9:**
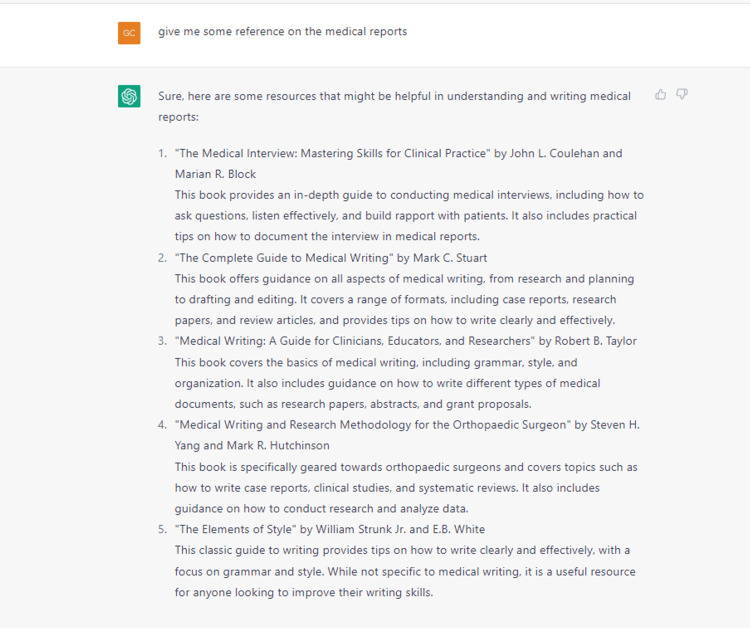
ChatGPT dialogue transcript-5

**Figure 10:**
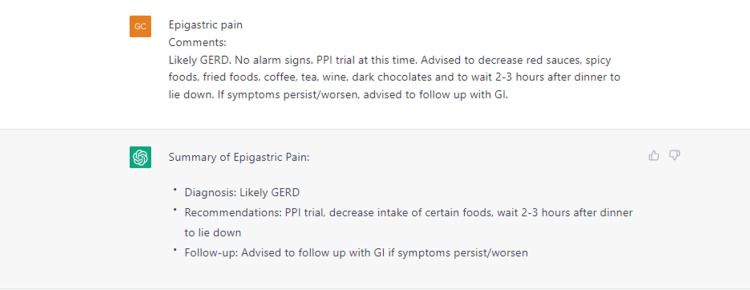
ChatGPT dialogue transcript-6

**Figure 11:**
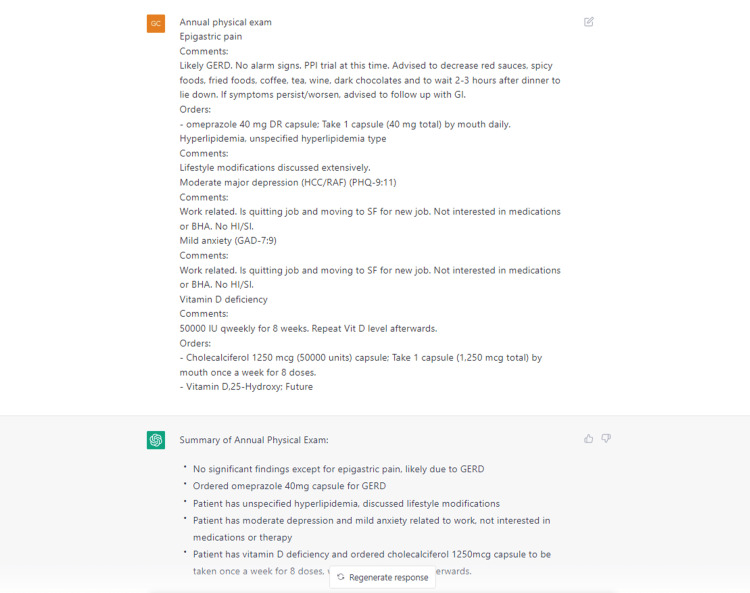
ChatGPT dialogue transcript-7

**Figure 12:**
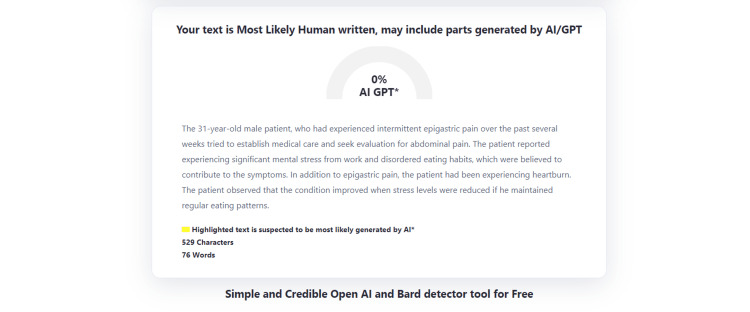
AI detection results with Zero-GPT analysis AI: Artificial intelligence; GPT: generative pre-trained transformer
